# Oral administration of interferon-α2b-transformed *Bifidobacterium longum *protects BALB/c mice against coxsackievirus B3-induced myocarditis

**DOI:** 10.1186/1743-422X-8-525

**Published:** 2011-12-08

**Authors:** Zhijian Yu, Zhen Huang, Chongwen Shao, Yuanjian Huang, Fan Zhang, Jin Yang, Lili Deng, Zhongming Zeng, Qiwen Deng, Weiseng Zeng

**Affiliations:** 1 Department of Infectious Diseases, the Affiliated Shenzhen Nanshan Hospital of Guangdong Medical College, No 89, Taoyuan Road, Nanshan district, 518052 Shenzhen, China; 2 Department of Cell Biology, Southern Medical University, No 1023, Satai Raod, Baiyun district, 510515 Guangzhou, China

**Keywords:** *Bifidobacterium*, Coxsackievirus B, Enterovirus, Interferon, Myocarditis, Oral administration

## Abstract

Multiple reports have claimed that low-dose orally administered interferon (IFN)-α is beneficial in the treatment of many infectious diseases and provides a viable alternative to high-dose intramuscular treatment. However, research is needed on how to express IFN stably in the gut. Bifidobacterium may be a suitable carrier for human gene expression and secretion in the intestinal tract for the treatment of gastrointestinal diseases. We reported previously that *Bifidobacterium longum *can be used as a novel oral delivery of IFN-α. IFN-transformed *B. longum *can exert an immunostimulatory role in mice; however the answer to whether this recombinant *B. longum *can be used to treat virus infection still remains elusive. Here, we investigated the efficacy of IFN-transformed *B. longum *administered orally on coxsackie virus B3 (CVB3)-induced myocarditis in BALB/c mice. Our data indicated that oral administration of IFN-transformed *B. longum *for 2 weeks after virus infection reduced significantly the severity of virus-induced myocarditis, markedly down regulated virus titers in the heart, and induced a T helper 1 cell pattern in the spleen and heart compared with controls. Oral administration of the IFN-transformed *B. longum*, therefore, may play a potential role in the treatment of CVB3-induced myocarditis.

## Introduction

The oral use of low doses of interferon (IFN)-α has been shown to exhibit beneficial effects in mice or human with acquired immunodeficiency syndrome (AIDS) [1], hepatitis B [2], aphthous stomatitis [3], and measles [4]. These studies have indicated that IFN-α/-β given orally provides a viable alternative to the current high-dose treatment intramuscularly [5,6]. The number of dairy and probiotic products that contain bifidobacteria has developed rapidly in recent years, and its probiotic properties have been extended further by the production of the recombinant *Bifidobacterium*-containing products [7-11]. Genetically engineered *Bifidobacterium *has been reported as a successful exogenous gene delivery carrier for the treatment of many diseases. We previously constructed a recombinant *B. longum *(IFN-transformed *B. longum*) that was inducible by arabinose to express efficiently secreted IFN-α2b *in vitro *[12]. Moreover, oral administration of IFN-transformed *B. longum *to mice increased intestinal sIgA and serum IFN-α2b levels, which suggested the potential clinical value of this bacterium as a kind of oral interferon in the treatment of virus infection [13].

Human IFN can inhibit coxsackievirus B3 (CVB3) replication *in vitro *and protects murine models from CVB3-induced myocarditis [14-19]. However, it is not clear whether oral administration of IFN can treat CVB3-induced myocarditis *in vivo*. In this study, the effect of IFN-transformed *B. longum *by oral administration on the development of CVB3-induced myocarditis in mice was evaluated.

## Materials and methods

### Cells and viruses

CVB3 (Nancy strain) was obtained from Prof. Wang at the Department of Biotechnology of Ginan University, China [20]. African green monkey kidney (Vero) cells were cultured in Dulbecco's modified Eagle's medium (DMEM) that contained 8% fetal calf serum (Gibco, Rockville, USA). Confluent cultures of Vero cells were infected with CVB3 and incubated at 37°C until an extensive cytopathic effect was observed (generally at 3-5 days post-infection). Subsequently, the culture media were collected, the cell debris was pelleted by centrifugation and removed and the supernatant was aliquoted and stored at-80°C.

### Bacteria culture

IFN-transformed *B. longum *were constructed by transforming *B. longum *with pBAD-SPIFN (BSPIFN) as reported [12]. Briefly, BSPIFN plasmids consisted of a fusion gene of the arabinosidase signal peptide and human IFN-α2b (hIFNα2b). Recombinant IFN-transformed *B. longum *contained an L-arabinose promoter and displayed highly efficient IFN-α2b expression [12]. The control plasmid-transformed *B. longum *bacteria were transformed with the control plasmid (pBAD-gIIIA) without the insertion of hIFN-α*2b *gene. Recombinant BSPIFN- and control plasmid-transformed *B. longum *were cultured anaerobically and prepared as described in our previous report [12]. A 10-μl suspension of bacteria was seeded onto BL agar plates (Nissui) that contained 100 g L^-1 ^ampicillin to determine the actual number of viable bacilli in the inocula. Colonies were counted after 24 h of anaerobic culture.

### Interventions and groups

This study was approved by the Ethics Committee of Southern Medical University (Guangzhou, China). Four-week-old male BALB/c mice (weight, 15 ± 0.5 g; Southern Medical University, USA) were inoculated i.p. with a 50% cell-culture infectious dose of CVB3 at 5 × 10^6 ^(as determined by plaque assay on Vero cells). Infected animals given this virus dosage survived for at least 6 months post-infection. We studied the efficacy of IFN-transformed *B. longum *on coxsackievirus B3-induced myocarditis. Forty BALB/c mice were inoculated with the virus and were divided into four groups. 'BIFN' group and 'Control' group animals were administered orally with IFN- and control plasmid- transformed *B. longum *for 2 weeks respectively after the inoculation of the virus. The 'IFN' group was injected i.m. with a therapeutic dose (1.5 μg kg^-^1 week^-1^) of pegylated IFN α2b (PegIntron). The 'saline' group was administered i.p. once daily with sterile saline after infection. Three mice were kept under the same conditions to act as the normal control. Recombinant bacteria were given to the mice orally once every 2 days using a tuberculin syringe attached to a 20-gauge olive-tip steel feeding tube, passed through the oral cavity and esophagus. All animals were killed at day 14 post-infection (following ether anesthesia). Up to day 14 post-infection, half of the murine hearts were dissected aseptically for virus titration and RNA extraction for cytokine quantity. The other half of the heart was used for hematoxylin-eosin (H&E) staining. The spleen was removed surgically to isolate mononuclear cells (MNCs).

### Morphometry

Hematoxylin-eosin staining was performed according to the standard techniques. The selected surfaces of myocarditis lesions studied were considered to be representative of the relative inflammatory area in the entire heart volume, because of the relatively homogeneous distribution of the myocarditis lesions in the affected hearts. The number of myocarditis lesions and their surface proportion were determined with H&E-stained sections of the hearts of the untreated and other groups. The proportion of the surface occupied by myocarditis lesions was determined by means of a conventional point-counting method, as reported earlier [21], by using an ocular grid that contained 121 equally spaced points. The surface proportion was taken to be an estimate of the percentage of heart tissue that was affected by focal myocarditis. Counting was performed on three sections per heart and the sections were evaluated at a magnification of less than ×200.

### Virus titration from heart homogenate

The aseptic hearts of the animals were weighed and homogenized in the minimal essential medium of 2 ml phosphate-buffered saline (PBS). The supernatant was subjected to three freeze-thaw cycles and centrifugation at 5000 rpm for 8 min, then was absorbed and diluted sequentially 10-fold in RPMI 1640 medium. Vero cells were grown to confluence in microtiter trays, infected with serial dilutions of the homogenates, and incubated for 3 h at 37°C. The cells were cultured for 72 h of cultivate, then the monolayers were fixed in 10% phosphate-buffered formalin and stained with crystal violet (Invitrogen); the numbers of plaques were counted. Virus titers were determined by standard plaque formation assay and expressed as per organ weight (in grams).

### Quantitation of transcript level of cytokines and Mx1

The total RNA of the heart tissues was extracted with Trizol reagent (Invitrogen, Carlsbad, CA, USA) and reverse transcribed into cDNA according to the manufacturer's protocol (Invitrogen, Carlsbad, CA, USA). Transcription of IFN-γ, TNF-α, Mx1 or the housekeeping gene β-actin was detected by real-time polymerase chain reaction (PCR) using a SYBR Green Master Mix (Applied Biosystems). Thermocycler conditions included an initial denaturation step at 94°C for 2 min; a three-step cycle procedure was carried out (denaturation, 94°C, 20 s; annealing, 58°C, 20 s; and extension, 72°C, 30 s) for 35 cycles. All reactions were performed in at least duplicate for each sample. Data were collected and analyzed quantitatively on an ABI Prism 7900 sequence detection system (Applied Biosystems). The β-actin gene was used as an endogenous control to normalize for differences in the amount of total RNA in each sample and the relative mRNA expression was calculate by normalization to the value of the β-actin transcripts. Primers for IFN-γ, TNF-α, Mx1 and the housekeeping gene β-actin have been reported previously [13,21].

### Preparation of mononuclear cells (MNCs) and cytokine detection

Spleens were removed surgically, and the MNCs were isolated as described previously [22]. Suspensions of MNCs from the spleen were prepared with RPMI-1640 culture medium that contained heat-inactivated fetal bovine serum (50 mL L^-1^), L-glutamine (2 mM), penicillin (1 × 10^5 ^U L^-1^), streptomycin (100 mg L^-1^), and HEPES (25 mM) (all from Life Sciences). The MNCs were seeded into 24-well plates (each well had 2 × 10^8 ^cells L^-1^) and stimulated subsequently with 100 μL of Con A (5 mg L^-1^) for 72 h at 37°C, 5% CO_2 _in air, and 95% humidity. The levels of IFN-γ, TNF-α and IL-10 in the supernatants were measured by OptEIA commercial enzyme-linked immunosorbent assay (ELISA) kits (BD Pharmingen), following the manufacturers' instructions. The detection limits of the ELISA assays were as follows: 2500 pg ml^-1 ^for IFN-γ, 825 pg ml^-1 ^for TNF-α and 650 pg ml^-1 ^for IL-10.

### Statistical analysis

Data were shown as the mean ± standard error of the mean (SEM). Statistical analyses of the data were performed by one-way analysis of variance (ANOVA), and the correlation between two variables was tested by bivariate correlation analysis using SPSS11.0; a *p*-value < 0.01 was considered to be statistically significant.

## Results

### Evaluations for the severity of myocarditis and virus replication

**Figure 1:**
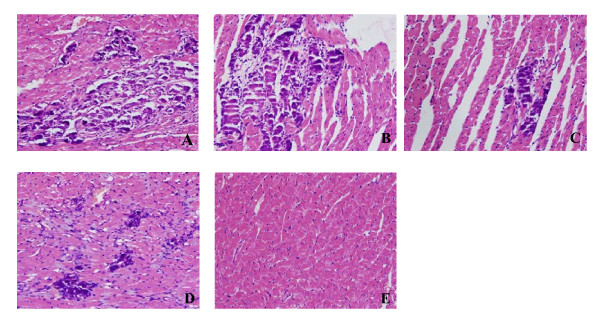
**Evaluation of the severity of myocarditis**. (**a-e**) are representative of histopathologic images in heart tissue from saline, B, BIFN and interferon (IFN) groups respectively (hemotoxylin and eosin (H&E) staining, original magnification × 200). Ten mice per group were analyzed in this study.

**Figure 2:**
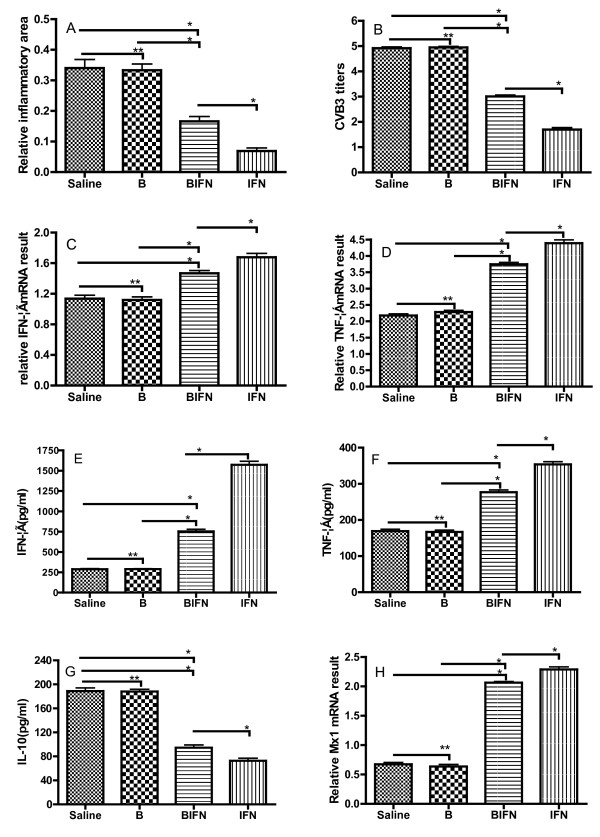
**Pathological areas of the heart**. The percentage of pathological areas in different groups was shown in (**a**). The coxsackievirus B3 (CVB3) titration detected by standard plaque formation assay was indicated in (**b**). The levels of interferon (IFN)-γ and tumor necrosis factor (TNF)-α mRNA were determined in (**c**) and (**d**) respectively. The concentration of, IFN-γ and TNF-α and were shown in (**e**, **f **and **g**) respectively. The detection of IFN-induced Mx1 mRNA was determined in (**h**). **versus B group, *p >*0.05; *versus BIFN group, *p *< 0.01.

The prominent cardiac inflammation area is observed in Figure 1. The percentage of the pathological area of the heart sections in the BIFN, B, IFN-α and saline groups was elevated and is compared in Figure 2a. The pathological area of heart sections in the BIFN group was significantly lower compared with the B and saline groups (*p *< 0.01) respectively, but markedly high compared with the IFN group. The levels of cardiac CVB3 titers and CVB3 RNA in the cardiac tissues of the BIFN group were significantly lower compared with the B and saline groups respectively and markedly higher compared with IFN group (*p *< 0.01) (Figure 2b).

### Enhanced levels of IFN-γ and TNF-α

We evaluated the T helper (Th) cell patterns induced by oral-administered IFN-transformed *B. longum *by measurement of the levels of two typical Th1 cytokines (IFN-γ and TNF-α). Our data showed that the cardiac IFN-γ and TNF-α mRNA levels in the BIFN group were enhanced significantly compared with that in the saline and B groups but were markedly reduced compared with that in the IFN-α group (*p *< 0.01; Figure 2c, d). Furthermore, we detected the levels of IFN-γ, TNF-α and IL-10 in the supernatant from the cultured MNCs from murine spleen. The levels of supernatant IFN-γ and TNF-α in the BIFN group were markedly raised compared with that in the saline and B groups (*p *< 0.01). Moreover, the levels of serum IL-10 in BIFN group were also markedly decreased compared with that in the saline and B groups (*p *< 0.01; Figure 2g).

### Increased expression of Mx1 mRNA in cardiac tissues

The Mx1 gene is induced typically by IFN. Its intracellular gene transcription level in a tissue samples can represent the relative amount of local type I IFN that stimulates the cells or tissues [23,24]. We measured the *Mx1 *gene transcription levels in cardiac tissues using real-time PCR to evaluate the local type I IFN concentration and activity. High Mx1 mRNA transcript levels were detected in the BIFN group compared with the saline and B groups respectively (*p *< 0.01; Figure 2h), a finding that was suggestive of a possibly high type I IFN concentration in this organ.

## Discussion

In this experiment, we proved the efficacy of the BIFN-transformed *B. longum *cells on the CVB3-induced myocarditis. Oral administration of IFN-transformed *B. longum *cells can reduce significantly the cardiac inflammatory area of CVB3-infected mice by day 14 compared with the B and saline groups respectively, which suggested that BIFN-transformed *B. longum *cells can improve the severity of disease. It has been demonstrated that the dominant pathogenic process in the early stages of CVB3 infection is the direct attack on myocardial cells by the virus, therefore antivirus treatment at this phase is very important to improve the development of virus infection [25]. Our data indicated that the cardiac virus titers in the murine heart of BIFN group were decreased significantly compared with B group, which indicated that this recombinant *B. longum *may improve cardiac inflammation partly by inhibition of virus replication at the early stage of CVB3 infection. Classical theories suggest that CD4^+ ^Th1 cells play a vital role against virus infection in adaptive immune responses by production of IFN-γ for effective clearance of virus invasion. In this study, IFN-transformed *B. longum *increased the expression of Th1 cytokines (IFN-γ and TNF-α) mRNA in cardiac tissue and enhanced the secretion of Th1 cytokines (IFN-γ and TNF-α) from splenocytes, which suggested that this recombinant *B. longum *is able to induce expression of CD4^+ ^Th1 cells against virus infection.

Our former studies have shown that hIFN-α2b from IFN-transformed *B. longum *is expressed mainly as a mature secretory cytokine and that serum hIFN-α2b level can be enhanced in the mice that have been administrated orally with *B. longum *[12,13]. As we know, the expression of hIFN-α2b in IFN-transformed *B. longum *is mainly induced by L-arabinose, which is a component of biopolymers such as hemicellulose and pectin [26]. The administration of IFN-transformed *B. longum *has been demonstrated to increase the serum and intestinal IFN-α2b level and we hypothesize that IFN expression by this bifidobacteria in mice might be induced persistently by L-arabinose in MRS or by the administered food and then enter the blood circulation by gastrointestinal absorption [13]. In this study, we compared mice either treated with saline and control *B. longum *mice respectively. The Mx1 mRNA levels, which represent the local tissue IFN concentration, were increased significantly in cardiac tissues in the BIFN group, which suggested that IFN-transformed *B. longum *can increase the level of active type 1 IFN locally. Further study is needed to ascertain how to control the expression of IFN stably in gut and whether these bacteria affect the microbial flora.

*Bifidobacterium *has many beneficial effects on human health that include prevention of infection, immunomodulation, promotion of lactose digestion and protection against colon cancer [9-11]. Recently, genetically engineered *Bifidobacterium *has been used successfully as an exogenous gene delivery carrier for bowel disease and cancer gene therapy [9-11,27]. This finding suggests that *Bifidobacterium *may be a suitable carrier for human gene expression and secretion in the intestinal tract for the treatment of gastrointestinal diseases. Here, we demonstrated the efficacy of BIFN-transformed *B. longum *to CVB3-induced myocarditis in the mice. Our data showed, compared with IFN-transformed *B. longum*, that IFN-α2b administered intramuscularly could reduce significantly virus infection, decrease the severity of virus-induced myocarditis, and induce a robust Th1 pattern in the spleen and heart. Nevertheless, IFN-transformed *B. longum *has its own advantages that include localization in the gastrointestinal cavity and spread of the physiological role locally [13]. Further experimentation is needed to evaluate whether IFN-transformed *B. longum *can be added to probiotic yogurt or diet and whether it can protect high-risk people who eat these products from the virus myocarditis. The model in this study was to inoculate with CVB3 intraperitoneally and this route may affect the therapeutic efficacy of IFN-transformed *B. longum *compared with IFN-α2b given intramuscularly. The preventive or therapeutic roles of IFN-transformed *B. longum *in virus diseases need to be studied further.

In conclusion, oral administration of IFN-transformed *B. longum *can decrease the severity of virus-induced myocarditis, reduce the virus titers in the heart and induce a Th1 pattern in the spleen and heart *in vivo*. IFN-transformed *B. longum *may play a potential role in the treatment of coxsackie virus B3-induced myocarditis. However, the advantages of the IFN-transformed *B. longum *in the treatment and prevention of enterovirus infection need to be studied further.

## Competing interests

The authors declare that they have no competing interests.

## Authors' contributions

QD and WZ conceived the study and QD wrote the paper. ZY, ZH, CS, YH, FZ, JY, LD and WZ participated in the laboratory studies. All authors read and approved the final manuscript.

## References

[B1] Katabira ET Sewankambo NK Mugerwa RD Belsey EM Mubiru FX Othieno C Kataaha P Karam M Youle M Perriens JH Lange JM Lack of efficacy of low dose oral interferon alfa in symptomatic HIV-1 infection: a randomised, double blind, placebo controlled trial Sex Transm Infect 1998 74 4 265 270 10.1136/sti.74.4.265 9924466 PMC1758122

[B2] Cummins J Beilharz M Krakowka S Oral use of interferon J Interferon Cytokine Res 1999 19 853 857 10.1089/107999099313352 10476928

[B3] Hutchinson VA Mok WL Angenend JL Cummins JM Richards AB Chronic major aphthous stomatitis: oral treatment with low-dose alpha-interferon Mol Biother 1990 2 4 217 220 2288721

[B4] Lecciones JA Abejar NJ Dimaano EE Bartolome R Cinco S Mariano N Yerro ME Cobar S Fuggan B A pilot double-blind, randomized, and placebo-controlled study of orally administered IFN-α-n1 (Ins) in pediatric patients with measles J Interferon Cytokine Res 1998 18 647 652 10.1089/jir.1998.18.647 9781802

[B5] Kim Y Thapa M Hua DH Chang KO Biodegradable nanogels for oral delivery of interferon for norovirus infection Antiviral Res 2011 89 2 165 173 10.1016/j.antiviral.2010.11.016 21144866 PMC3027895

[B6] Dec M Puchalski A Use of oromucosally administered interferon-alpha in the prevention and treatment of animal diseases Pol J Vet Sci 2008 11 175 186 18683548

[B7] Hu B Kou L Li C Zhu LP Fan YR Wu ZW Wang JJ Xu GX *Bifidobacterium longum *as a delivery system of TRAIL and endostatin cooperates with chemotherapeutic drugs to inhibit hypoxic tumor growth Cancer Gene Ther 2009 16 655 663 10.1038/cgt.2009.7 19229287

[B8] Reyes Escogido ML De León Rodríguez A Barba de la Rosa AP A novel binary expression vector for production of human IL-10 in *Escherichia coli *and *Bifidobacterium longum* Biotechnol Lett 2007 29 1249 1253 10.1007/s10529-007-9376-8 17487549

[B9] Shkoporov AN Efimov BA Khokhlova EV Kafarskaia LI Smeianov VV Production of human basic fibroblast growth factor (FGF-2) in *Bifidobacterium breve *using a series of novel expression/secretion vectors Biotechnol Lett 2008 30 1983 1988 10.1007/s10529-008-9772-8 18575808

[B10] Tang W He Y Zhou S Ma Y Liu G A novel *Bifidobacterium infantis*-mediated TK/GCV suicide gene therapy system exhibits antitumor activity in a rat model of bladder cancer J Exp Clin Cancer Res 2009 28 155 10.1186/1756-9966-28-155 20015348 PMC2803447

[B11] Yazawa K Fujimori M Nakamura T Sasaki T Amano J Kano Y Taniguchi S *Bifidobacterium longum *as a delivery system for gene therapy of chemically induced rat mammary tumors Breast Cancer Res Treat 2001 66 165 170 10.1023/A:1010644217648 11437103

[B12] Deng Q Zeng W Yu Z Signal peptide of Arabinosidase enhances secretion of interferon-alpha2b protein by *Bifidobacterium longum* Arch Microbiol 2009 191 681 686 10.1007/s00203-009-0496-5 19652954

[B13] Yu Z Zeng Z Huang Z Lian J Yang J Deng Q Zeng W Increased mRNA expression of interferon-induced Mx1 and immunomodulation following oral administration of IFN-α2b-transformed *B. longum *to mice Arch Microbiol 2010 192 633 638 10.1007/s00203-010-0589-1 20535450

[B14] Heim A Grumbach I Pring-Akerblom P Stille-Siegener M Muller G Kandolf R Figulla HR Inhibition of coxsackievirus B3 carrier state infection of cultured human myocardial fibroblasts by ribavirin and human natural interferon-alpha Antivir Res 1997 34 101 111 10.1016/S0166-3542(97)01028-0 9191017

[B15] Kandolf R Canu A Hofschneider PH Coxsackie B3 virus can replicate in cultured human fetal heart cells and is inhibited by interferon J Mol Cell Cardiol 1985 17 167 181 10.1016/S0022-2828(85)80019-5 3889351

[B16] Okada I Matsumori A Matoba Y Tominaga M Yamada T Kawai C Combination treatment with ribavirin and interferon for coxsackievirus B3 replication J Lab Clin Med 1992 120 569 573 1328433

[B17] Padalko E Nuyens D De Palma A Verbeken E Aerts JL De Clercq E Carmeliet P Neyts J The interferon inducer ampligen [poly(I)-poly(C12U)] markedly protects mice against coxsackie B3 virus-induced myocarditis Antimicrob Agents Chemother 2004 48 1 267 274 10.1128/AAC.48.1.267-274.2004 14693549 PMC310159

[B18] Deonarain R Cerullo D Fuse K Liu PP Fish EN Protective role for interferon-beta in coxsackievirus B3 infection Circulation 2004 110 3540 3543 10.1161/01.CIR.0000136824.73458.20 15249500

[B19] Wang YX da Cunha V Vincelette J White K Velichko S Xu YF Gross C Fitch RM Halks-Miller M Larsen BR Antiviral and myocyte protective effects of murine interferon-beta and -alpha2 in coxsackievirus B3-induced myocarditis and epicarditis in BALB/c mice Am J Physiol Heart Circ Physiol 2007 293 H69 H76 10.1152/ajpheart.00154.2007 17434974

[B20] Wang YF Wang XY Ren Z Qian CW Li YC Kaio K Wang QD Zhang Y Zheng LY Jiang JH Yang CR Liu Q Zhang YJ Phyllaemblicin B inhibits coxsackie virus B3 induced apoptosis and myocarditis Antiviral Res 2009 84 2 150 158 10.1016/j.antiviral.2009.08.004 19699238

[B21] Yuan J Yu M Lin QW Cao AL Yu X Dong JH Wang JP Zhang JH Wang M Guo HP Cheng X Liao YH Th17 cells contribute to viral replication in coxsackievirus B3-induced acute viral myocarditis J Immunol 2010 185 7 4004 4010 10.4049/jimmunol.1001718 20802148

[B22] Alignani D Maletto B Liscovsky M Rópolo A Morón G Pistoresi-Palencia MC Orally administered OVA/CpG-ODN induces specific mucosal and systemic immune response in young and aged mice J Leukocyte Biol 2005 77 898 905 10.1189/jlb.0604330 15758079

[B23] Bollati-Fogolín M Müller W Virus free, cell-based assay for the quantification of murine type I interferons J Immunol Methods 2005 306 169 175 10.1016/j.jim.2005.08.005 16209875

[B24] Petry H Cashion L Szymanski P Ast O Orme A Gross C Bauzon M Brooks A Schaefer C Gibson H Qian H Rubanyi GM Harkins RN Mx1 and IP-10: biomarkers to measure IFNbeta activity in mice following gene-based delivery J Interferon Cytokine Res 2006 26 699 705 10.1089/jir.2006.26.699 17032164

[B25] Dennert R Crijns HJ Heymans S Acute viral myocarditis Eur Heart J 2008 29 2073 2082 10.1093/eurheartj/ehn296 18617482 PMC2519249

[B26] David RL CRC Handbook of Chemistry and Physics 2007 88 Boca Raton: CRC Press 110

[B27] Yao J Wang JY Lai MG Li YX Zhu HM Shi RY Mo J Xun AY Jia CH Feng JL Wang LS Zeng WS Liu L Treatment of mice with dextran sulfate sodium-induced colitis with human interleukin 10 secreted by transformed *Bifidobacterium longum* Mol Pharm 2011 8 2 488 497 10.1021/mp100331r 21271712

